# Pathogenesis and Nomenclature of Odontogenic Carcinomas: Revisited

**DOI:** 10.1155/2014/197425

**Published:** 2014-03-30

**Authors:** Swagatika Panda, Sujit Ranjan Sahoo, Gunjan Srivastav, Subrat Padhiary, Kanika Singh Dhull, Sonia Aggarwal

**Affiliations:** ^1^Department of Oral Pathology and Microbiology, Institute of Dental Sciences, Kalinga Nagar, Bhubaneswar 751003, India; ^2^Department of Prosthodontics, Institute of Dental Sciences, Kalinga Nagar, Bhubaneswar 751003, India; ^3^Department of Oral and Maxillofacial Surgery, Institute of Dental Sciences, Kalinga Nagar, Bhubaneswar 751003, India; ^4^Department of Pedodontics, Kalinga Institute of Dental Sciences, Bhubaneswar 751024, India; ^5^Department of Conservative Dentistry, Institute of Dental Sciences, Kalinga Nagar, Bhubaneswar 751003, India

## Abstract

Odontogenic carcinoma is rare group of malignant epithelial odontogenic neoplasms with characteristic clinical behavior and histological features, which requires an aggressive surgical approach. The pathogenesis of this rare group remains still controversial and there have been many varied opinions over the classification of this rare group of lesions. As there have not been many reviews on odontogenic carcinoma, the existing knowledge is mostly derived from the published case reports. This review is discussing the pathogenetic mechanisms and is updating the knowledge on nomenclature system of less explored odontogenic carcinomas. This review might throw light on the pathogenesis and nomenclature system of odontogenic carcinoma and this knowledge may be applied therapeutically.

## 1. Introduction 

Odontogenic tumours are broadly classified into benign and malignant odontogenic tumours. Odontogenic carcinomas are the malignant epithelial odontogenic neoplasms which comprise the first category of the 2005 WHO classification of odontogenic tumours [1]. These tumours are believed to take origin from the epithelial components of the odontogenic apparatus. The cell rests of Malassez, reduced enamel epithelium, the rests of Serres in the gingiva, and the linings of odontogenic cysts represent the precursor cells for odontogenic carcinoma. There has been involvement of several genes and the underlying mechanisms for cancer specific genes include a range of functional activities: (1) transcription, (2) signaling transduction, (3) cell-cycle regulation, (4) apoptosis, (5) differentiation, and (6) angiogenesis. These lesions are usually locally aggressive with radical surgery being the primary mode of treatment. Because of their rarity, much of the existing information about malignant odontogenic tumors with regard to their nomenclature, pathogenesis, clinicopathological features, biological behaviour, and therapeutics is derived from case reports or small series. We hereby present a review on odontogenic carcinomas focusing on its nomenclature systems and pathogenesis.

## 2. Classification 

In 1971, the World Health Organization (WHO) [2] published its classification of odontogenic carcinomas recognizing the subtypes (Table 1). In 1982, Elzay [3] opined that the WHO classification does not accommodate tumours that are histologically identical to classic ameloblastoma and metastasize from ameloblastoma-like lesions that are histologically malignant before metastasizing. He proposed a modification of the classification in which all primary intraosseous carcinomas (PIOCs) that do not involve the salivary glands would be classified as PIOCs, which would then be subclassified (Table 2). Elzay [3] suggested that all intraosseous carcinomas fulfilling the above criteria be classified under the general heading of PIOC and then be subclassified and subtyped according to histologic evidence of origin.

In 1984, Slootweg and Müller [4] further emphasized that ameloblastomas may exhibit malignant features other than metastasis and suggested a modified classification system (Table 3) for malignant tumours with features of ameloblastoma, based on characteristics of malignancy.

Elzay [3] and Slootweg and Müller [4] used the term ameloblastic carcinoma to convey the presence of cytologic features of malignancy. The degree of differentiation in epithelial neoplasms is usually considered to be significant in predicting biologic behaviour of metastasis. The main difference between Elzay's and Slootweg and Müller's schemes relates to the minor point of histogenesis. According to these authors, the term ameloblastic carcinoma should be used to designate lesions that exhibit histologic features of both ameloblastoma and carcinoma [3–5]. The tumour may metastasize and histologic features of malignancy may be found in either the primary tumour, the metastases, or both [4–6]. The term malignant ameloblastoma should be confined to those ameloblastomas that metastasize despite an apparently typical benign histology in both the primary and the metastatic lesions [7–10]. Kruse et al. in 2009 [11] proposed another classification system for ameloblastic carcinomas (Table 4). A significant disadvantage, however, remains the presupposition that the origin, including the histopathogenesis of ameloblastic carcinoma, is still unknown. Zarbo et al. [12] documented a spindle cell variant of malignant ameloblastoma.

Carcinomas associated with ameloblastoma have had several terminologies within the medical literature, thus posing a problem in accurately separating malignant ameloblastoma from ameloblastic carcinoma. Several authors have attempted to make a distinction between these two entities because ameloblastic carcinoma is clinically more aggressive. These definitions include a well-differentiated ameloblastoma with histologically malignant epithelial component; a tumor with histologic evidence of malignancy and features of ameloblastoma and concomitant squamous cell carcinoma; a tumor with combined features of an ameloblastoma with less differentiated areas; and any ameloblastoma with histologic evidence of malignancy in the primary tumor or the recurrent tumor, irrespective of whether the tumor has metastasized [13]. It is classified as primary type; secondary type, intraosseous; secondary type; and peripheral type according to the WHO classification of 2005 (Table 5) [14]. Histological subtypes of ameloblastic carcinoma have been suggested by Kamath et al. [15] (Table 6).

Primary intraosseous carcinoma (PIOC) is a carcinoma arising within the jaw. It was first described by Loos in 1913 as central epidermoid carcinoma of the jaw. The term PIOC was coined by Pindborg et al. in 1971 [2]. It is derived either from the remnants of odontogenic epithelium, epithelial rests of Malassez, or remnants of dental lamina [16, 17]. WHO defines PIOC as “A Squamous cell carcinoma arising within the jaw, having no initial connection with the oral mucosa and presumably developing from residues of the odontogenic epithelium.” Hence the tumor is also ambiguously referred to as odontogenic carcinoma [18].

The WHO classification dearly separates this entity from malignant ameloblastoma and other carcinomas arising from odontogenic cysts. Moreover, squamous cell carcinoma involving the jaws as an extension of carcinoma from either the gingival, alveolar ridge, floor of mouth, and maxillary sinus or via metastases from a distant site is excluded. The replacement of the word alveolar by osseous in WHO terminology seems reasonable as alveolar is an anatomic term relating specifically to bony area of the jaws adjacent to the teeth. Since most of the central carcinomas reported in the jaws are not confined to alveolar area, the term osseous is less restrictive and correct [19].

The clear cell odontogenic tumor is described as a benign but locally invasive odontogenic tumor in the current World Health Organization classification for odontogenic tumors. However, recent data on this variant have all indicated aggressive behaviour characterized by an infiltrative growth pattern with a high rate of recurrence and local or distant metastasis. High mortality rates are reported to occur due to tumour progression. Consequently, the name clear cell odontogenic carcinoma was thought to be more appropriate in view of the malignant potential manifested by this neoplastic lesion [20–22].

Ghost cell odontogenic carcinoma (GCOC) was first described in detail as a malignant focus within a calcifying odontogenic cyst (COC) by Ikemura et al. in 1985 [23]. Recently, according to the World Health Organization (WHO) classification, COC was recategorized as a calcifying cystic odontogenic tumor (CCOT), and GCOC was defined as a malignant odontogenic epithelial tumor with features of CCOT and/or dentinogenic ghost cell tumor (DGCT) [24].

## 3. Malignant Ameloblastoma 

Metastasizing ameloblastoma ambiguously termed as malignant ameloblastoma is “a neoplasm in which the features of an ameloblastoma are shown by the primary growth in the jaws and by any metastatic growth.” Basically this definition proves the presence of metastases, with histologic features having a minor role in diagnosis. The diagnosis of malignant ameloblastoma can only be made after the occurrence of metastatic deposits. This definition profoundly differs from the definition given by WHO which says that malignant ameloblastoma is a neoplasm in which pattern of ameloblastoma is combined with cytological features of malignancy [25]. This lack of histologic delineation has caused much confusion, as demonstrated by the fact that the tumours consisting exclusively of conventional well-differentiated ameloblastoma in primary as well as in metastatic lesions and tumours displaying a more anaplastic morphology in both the primary and metastatic growths have been classified as malignant ameloblastoma. However, Kruse et al. [11] have solved the issue to some extent by subclassifying malignant ameloblastoma into two types, (a) metastasis with features of an ameloblastoma (well differentiated) and (b) metastasis with malignant features (poorly differentiated).

According to Okada et al., differences actually do exist in biologic behavior of malignant ameloblastoma and metastasizing ameloblastoma. Most malignant ameloblastomas do not arise de novo, but rather represent the malignant transformation which takes about 10 years to develop malignancy. The latter may occur spontaneously or due to miscellaneous factors. Furthermore, it is suggested that most metastasizing ameloblastomas take several years to metastasize. Hence, the difference in average age of the patients suffering from conventional ameloblastoma and malignant ameloblastoma seems to be based on these time lags [26].

Though cytologic atypia is not a feature of malignant ameloblastoma, spindling of the cells is recognized in some solid proliferating areas which seems to be one of the peculiar characteristics of malignant ameloblastoma. Ultrastructural studies of spindle cell variant of malignant ameloblastoma [12] have shown that these cells have only few desmosomes, which suggest that they are loosely attached to each other, more so in spindle cell areas. In addition, the basal lamina does not clearly surround the cell nests [26]. Hartman found that metastatic ameloblastomas often present the granular cell variant [27]. Even though the presence of granular cells in ameloblastoma is infrequent, the observation that it accounts for so many cases of malignant disease may be significant.

There are several factors that appear to be contributory to the development of metastatic disease, including extensive local disease, duration of the primary tumour, frequent surgical procedures, radiotherapy and chemotherapies, and mandibular focus of the disease [28–30]. In case of metastasis from ameloblastoma following three routes are suggested, that is, hematogenous, lymphatic route, and by aspiration [29]. Most cases of malignant ameloblastoma to the lungs appear to be associated with hematogenous route. This is supported by the fact that tumour foci are often found diffusely scattered bilaterally and clusters of tumour cells are often seen to be closely associated with surrounding blood vessels. Further, the review of the literature shows that metastatic lesions to lung are often seen bilaterally and with multiple nodules [31]. This would lend support to the theory of hematogenous spread. On the other hand, based on the fact that tumour casts are often found in the bronchi and bronchioles, some authors support the theory that metastatic spread occurs through aspiration of tumour contents. In support to this theory, these authors have cited the fact that metastatic deposits are often located primarily in right lung or bilaterally present [29]. According to Houston et al., during embryogenesis, the odontogenic epithelium becomes entrapped in lymphoid tissue. When this epithelium undergoes benign neoplastic changes, an ameloblastoma could develop within a lymph node [32].

## 4. Ameloblastic Carcinoma 

**Figure 1:**
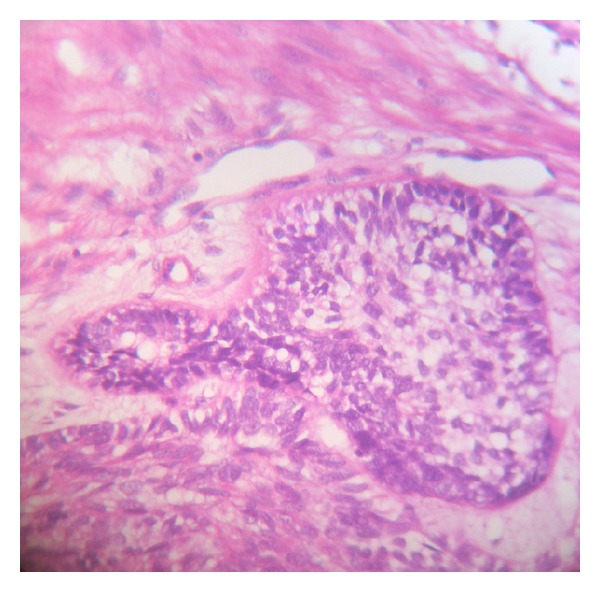
H&E stained section of ameloblastic carcinoma showing morphology of ameloblastoma with nuclear hyperchromatism along with pleomorphism and localised basaloid hyperplasia.

The term ameloblastic carcinoma (AC) has recently been introduced to describe ameloblastomas in which there was histologically malignant transformation in association with less-differentiated metastatic growth, in other words, to describe tumours that show features of ameloblastoma intermingled with those of carcinoma [4]. These lesions exhibit cytologic and/or histologic evidence of malignancy (Figure 1), regardless of whether they have metastasized [5].

Chromosomal imbalances in ameloblastomas are reported to be rare, with losses in chromosomes 22 and 10 being most frequent. Aneuploidy is more common in AC and may predict malignant potential [33].

In ameloblastomas including its peripheral variant, ameloblastomatous epithelium preserves the capacity for synthesis and incorporation of laminin within the basement membrane substance. For the cancer cells available evidence now indicates that carcinomas as well as their normal counterpart do synthesize laminin, but they fail to incorporate the product into an insoluble phase of their basement membrane. This has also been reported in regard to ameloblastic carcinoma [34].

Most of the reported cases of ACs arise de novo; however, in some instances, a preexisting benign ameloblastoma after several recurrences developed a malignant phenotype. The transformed tumour may continue to show features of ameloblastoma with concomitant dysplastic cytologic features or the recurrences may represent squamous cell carcinoma. These transformed cases are termed dedifferentiated ameloblastomas [27].

The rarest variant of ameloblastic carcinoma is the peripheral ameloblastic carcinoma that arises from the gingival or alveolar mucosal epithelium. It is an extremely rare odontogenic tumor derived from the remnants of dental lamina and/or mucosal epithelium of the oral mucosa. The varied cytopathologic findings may be related to the proliferation and transformation of basal cells of the mucosal epithelium toward ameloblastic carcinoma and variable squamous differentiation [35].

Slater has mentioned the term “spindle-shaped ameloblastic carcinoma” in 1999. Describing a group of odontogenic carcinomas that showed a spindle cell sarcomatoid change, he advocated differentiation of these lesions from odontogenic sarcomas by the absence of ameloblastic fibrosarcoma-like features in the spindle cell variant of ameloblastic carcinoma. In addition, the use of immunohistochemical markers served to highlight the nonsarcomatous origin of the spindle cells [36].

Spindle-shaped cells in a malignant lesion can be characterized as malignant fibroblasts (MF) or cancer-associated fibroblasts (CAF). Although many are utilized, presently there are no diagnostic markers for any of the above-mentioned cells. According to criteria set by de Wever et al. [38], a spindle cell is characterized as stromal MF if it is positive to alpha-SMA and to at least three other markers from a list of positive markers such as paladin 4Ig, podoplanin, vimentin/desmin, endosialin, and cadherin 11, prolyl-4 hydroxylase (P4H), as well as negative to markers such as cytokeratin, CD14, CD31, CD34, and smoothelin. It is regarded as CAF if this criterion is not met. Bello et al. [39] have stated that expression of alpha SMA in the epithelial odontogenic islands is virtually diagnostic of a spindle-shaped variant of AC.

In genome analysis, the CpG methylation of p16 (cyclin-dependent kinase inhibitor 2A) is observed in all ameloblastic carcinoma samples, but only one ameloblastoma specimen exhibits the mutation. Therefore, it is presumed that p16 alteration may play a role in the malignant progression of ameloblastic carcinoma [40]. More recently, 5q13 amplification was demonstrated by comparative genomic hybridization (CGH) in an AC [41]. Recently Siriwardena et al. have suggested the role of aberrant *β*-catenin expression and adenomatous polyposis coli gene mutation in AC [42].

## 5. Squamous Cell Odontogenic Carcinoma 

Squamous cell odontogenic carcinomas (WHO1.2.1) are subdivided into 3 subcategories: (1) primary intraosseous carcinomas (WHO1.2.1.2)—solid, (2) carcinomas arising from epithelial lining of odontogenic cyst, and (3) carcinomas arising from benign epithelial odontogenic tumour like KCOT. The exclusionary diagnosis of primary intraosseous squamous cell carcinoma of the mandible is made only after no distant primary site is identified 6 months after treatment [1].

On analyzing 32 cases of squamous cell odontogenic carcinoma, Eversole has reported that 75% were associated with teeth [43]. A retrospective study of 116 cases of PIOSCC between 1938 and 2010 showed that there have been only 16 known cases of PIOSCC arising from KCOT [44].

Alevizos et al. [37] have suggested the genetic expression profiling and genes that are upregulated and downregulated in odontogenic carcinomas (Table 7). Genes with a greater than threefold upregulation and downregulation in the squamous cell OC compared with oral mucosal squamous cell carcinoma. Aberrant *β*-catenin expression and adenomatous polyposis coli gene mutation were proven by authors [45].

## 6. Primary Intraosseous Carcinoma (PIOC)

The diagnosis of PIOC is often difficult as the lesion must be differentiated from carcinomas that may invade the bone from the overlying soft tissues or from the tumors that have metastasized to the jaw from a distant site [46]. Review of the literature showed that the origin of PIOC varies as it may arise from reduced enamel epithelium or even odontogenic cysts [47, 48].

Lucas [49], in commenting on the cells of origin of central carcinoma of the jaws, presumed that carcinoma could arise from odontogenic cell rests or from enclaved epithelium at the site of embryonic fissures. Investigators now believe that embryonic fissures have little or no role in the development of the jaws. However, remnants of epithelium do persist in the area of the incisive canal, and cases have been reported in this location. The fact that intraosseous carcinomas are rare in bones other than the jaws supports the concept that primary intraosseous carcinomas are odontogenic in origin. The most likely source of malignant epithelium in the jaws originates from the infoldings into the jaws of oral epithelium destined to become odontogenic. If one considers the age, location, and odontogenic origin of the reported cases of primary intraosseous carcinoma, it seems plausible to question whether or not some cases may represent squamous cell carcinoma arising in a previously existing ameloblastoma.

It is characterized by islands of neoplastic squamous epithelium with the features of squamous cell carcinoma. Most lesions are moderately differentiated without prominent keratinization. The stroma may or may not exhibit an inflammatory infiltrate.

### 6.1. Primary Intraosseous Squamous Cell Carcinoma Derived from Keratocystic Odontogenic Tumour

This is rather explained as a squamous cell carcinoma arising within the jaws without connection to the oral mucosa in the presence of a keratocystic odontogenic tumour (KCOT). The histological appearance of this lesion is typically that of a keratinizing well-differentiated squamous cell carcinoma in conjunction with KCOT. The main differential diagnosis would include keratoameloblastoma, squamous odontogenic tumour, central high-grade mucoepidermoid carcinoma, and metastatic lesions [50].

### 6.2. Primary Intraosseous Squamous Cell Carcinoma Derived from Odontogenic Cysts

Histopathologically this tumour is characterized as a cyst lined by any type of epithelium that can be seen in odontogenic cysts in association with a squamous cell carcinoma. Various degrees of dysplasia may be observed in the epithelial cyst lining. Secondary involvement of a cyst by an adjacent carcinomatous lesion and cystic degeneration in a primary intraosseous carcinoma has to be excluded before ascertaining the diagnosis of carcinoma arising in odontogenic cyst. In 1975, Gardner proposed the following criteria for diagnosis of carcinoma arising in an odontogenic cyst [51, 52]:

1. a microscopic transition area from benign cystic epithelial lining to invasive SCCA,
2. no carcinomatous changes in the overlying epithelium,
3. no source of carcinoma in the adjacent structures.

Waldron and Mustoe [53] have added a 4th criterion which says that the possibility of a metastatic tumour must be ruled out by physical and radiological examination and the subsequent clinical course.

## 7. Clear Cell Odontogenic Carcinoma 

**Figure 2:**
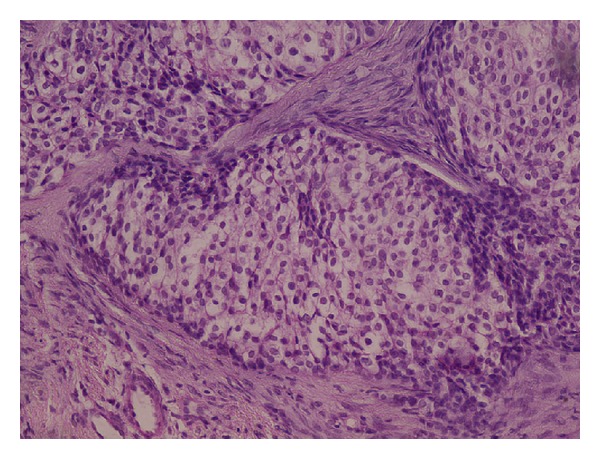
H&E stained section of clear cell odontogenic carcinoma showing polygonal clear cells with peripheral hyperchromatic columnar cells with reversal of basal cell polarity and localized basal cell hyperplasia.

Histologically, clear cell odontogenic carcinoma (CCOC) consists of clear cells (Figure 2), which are positive for cytokeratin and negative for vimentin and also negative for mucicarmine, which differentiates it from some of the other clear cell tumors such as mucoepidermoid carcinoma and renal carcinoma and calcifying epithelial odontogenic tumor (CEOT) [54].

Tumors with a conspicuous clear cell component in the head and neck region can impose serious problems with respect to differential diagnosis. They can originate from various sources, including salivary gland tumors, metastatic renal cell carcinoma, melanotic tumors, and other odontogenic tumors, such as ameloblastoma and CEOT [55].

According to an IHC study done by Li et al. [55], in CCOC, most of the clear cells contained diastase-digestible, PAS-positive granules, whereas none of the tumor cells stained with Alcian blue, indicating the presence of glycogen rather than mucin within the cytoplasm. Negative Congo red reactivity indicated that the hyaline osteoid/dentinoid structures in the lesion were different from amyloid deposits. Immunocytochemically, the tumor cells showed positive staining for wide-spectrum cytokeratin, CK-19, and epithelial membrane antigen, but negative staining for vimentin, S100 protein, desmin, smooth muscle actin, human melanoma antigen (HMB-45), and *α*1-antichymotrypsin [56]. Expression of cytokeratin and epithelial membrane antigen has been assessed in various odontogenic lesions [57], and CK-19 has been shown to react with all kinds of odontogenic epithelial cells [58]. In salivary glands and their tumors, however, only ductal cells exhibit focal expression of CK-19. Thus, the immunocytochemical profile of the CCOC suggests that they are of odontogenic epithelial origin. In addition, the presence of eosinophilic, hyaline, fibrillar, and dentin/bonelike structures between tumor cell nests and fibrous stroma also suggests that some of the tumors possess epithelial-mesenchymal inductive capacity, a feature shared by many odontogenic epithelial tumors [58].

Expressions of Msx and Dlx homeobox genes have been studied by Ruhin-Poncet et al. in various odontogenic tumors including CCOC [59]. Dlx2 and Dlx3 are expressed during tooth morphogenesis and have been shown to play a key role during cell differentiation and apoptosis. This study showed a lack of Dlx2, Dlx3, Msx2, and Bmp2 expression, specifically in CCOC, compared with ameloblastomas and the normal situation.

Dysregulation in Bmp signaling is suggested in a study by the evident absence of Bmp2 transcript expression in the CCOC and not of Bmp4 transcripts. Bmp2 is downregulated at the time of terminal differentiation of ameloblasts, suggesting that the differentiation process is affected in the CCOC cells. Bmp2 not only stimulates expression of Msx1 and Msx2, but also induces Dlx2 expression [60] and transactivates Dlx3. The lack of Bmp2 may be responsible for the absence of these two homeobox genes in the case of CCOC. DNA analysis has shown a polyploid population with DNA index of 1.93 and an S-phase of 10.2% [61]. Comparative genomic hybridization discloses consistent chromosomal aberrations in both primary and metastatic CCOC [62].

## 8. Ghost Cell Odontogenic Carcinoma 

Based on study of Kodama et al. [63], four basic different pathogenic mechanisms can be suggested which could lead to following subtypes of ghost cell odontogenic carcinoma, that is, type 1, de novo (not associated with preceding DGCT or CCOT), representing a secondary onset from an undiagnosed primary lesion; type 2, GCOC arising secondary to a benign CCOT; type 3, arising from DGCT; and type 4,  arising from any other odontogenic cyst or odontogenic tumour. Histopathology aids in the final diagnosis of the lesion. It reveals many malignant epithelial islands in a background of fibrous stroma. Ghost cells are a prominent feature in the epithelial islands similar to that of calcifying cystic odontogenic tumour. Dysplastic dentin may be present [64]. Immunohistochemical overexpression of p53 protein as well as PCNA is demonstrated in the tumour cells [65, 66].

## 9. Conclusion 

Odontogenic carcinoma though very aggressive lesion have been explored very less. This review might throw light on the pathogenesis and nomenclature system of odontogenic carcinoma and this knowledge may be applied therapeutically. In particular, there should be future research directed to pathogenesis of ghost cell odontogenic carcinoma and malignant granular cell odontogenic tumour.

## Figures and Tables

**Table 1:** 1971 WHO classification of odontogenic carcinomas [2].

Types	Odontogenic carcinomas
1	Malignant ameloblastoma
2	Primary intraosseous carcinoma
3	Other carcinomas arising from odontogenic epithelium, including those arising from odontogenic cysts

**Table 2:** Elzay's classification of ameloblastic carcinomas [3].

Types	Ameloblastic carcinomas
1	Arising from an odontogenic cyst
2	Arising from an ameloblastoma a: well differentiated (malignant ameloblastoma) b: poorly differentiated (ameloblastic carcinoma)
3	Arising de novo a: nonkeratinizing b: keratinizing

**Table 3:** Slootweg and Müller's classification of ameloblastic carcinomas [4].

Types	Ameloblastic carcinomas
1	Primary intraosseous carcinoma ex odontogenic cyst
2	Arising from ameloblastoma a: malignant ameloblastoma b: ameloblastic carcinoma, arising de novo, ex ameloblastoma, or ex odontogenic cyst
3	Primary intraosseous carcinoma de novo a: nonkeratinizing b: keratinizing

**Table 4:** Classification of ameloblastic carcinomas by Kruse et al. in 2009 [11].

Types	Ameloblastic carcinomas
1	Malignant ameloblastoma a: metastases with features of an ameloblastoma (well differentiated) b: metastases with malignant features (poorly differentiated)
2	Ameloblastic carcinoma arising from an ameloblastoma a: without metastases (malignant ameloblastoma) b: metastases with features of an ameloblastoma (well differentiated) c: metastases with malignant features (poorly differentiated)
3	Ameloblastic carcinoma with unknown origin histology (de novo) a: without metastases b: metastases with features of an ameloblastoma (well differentiated) c: metastases with malignant features (poorly differentiated)

**Table 5:** 2005 WHO classification of odontogenic carcinomas [1].

Types	Odontogenic carcinomas
9310/3	Metastasizing (malignant) ameloblastoma
9270/3	Ameloblastic carcinoma—primary type 9270/3: ameloblastic carcinoma—secondary type (dedifferentiated), intraosseous 9270/3: ameloblastic carcinoma—secondary type (dedifferentiated), peripheral 9270/3: primary intraosseous squamous cell carcinoma—solid type 9270/3: primary intraosseous squamous cell carcinoma derived from keratocystic odontogenic tumour 9270/3: primary intraosseous squamous cell carcinoma derived from odontogenic cysts
9341/3	Clear cell odontogenic carcinoma
9302/3	Ghost cell odontogenic carcinoma

**Table 6:** Histological subtypes of ameloblastic carcinomas by Kamath et al. [15].

Subtypes	Characteristic histological features
(1) Ameloblastic type	With atypical and pleomorphic ameloblasts
(2) Granular cell type	Majority of cells are of the granular cell variety
(3) Clear cell variant	Majority of cells are of the clear cell variety
(4) Spindle cell variant	Spindle cell differentiation predominates the histology

**Table 7:** Genetic profiling of odontogenic carcinomas by Alevizos et al. [37].

Upregulated genes	Downregulated genes
(1) Nuclear factor (Nf-116) (2) Epidermal keratin type II (3) MEF2C transcription factor (4) Metalloproteinase (5) Tyrosine phosphatases CIP 2 (6) Transforming growth factor beta binding protein (7) Mitogen inducible gene 2 (8) Oncofetal antigen 5T4	(1) Epidermal keratin types 1, 13, 15, and 16 (2) Transforming growth factor beta 3 receptor (3) Differentiation dependent A4 protein (4) Ribosomal proteins L3, L8, L28, L31, and L35 (5) ARF activated phosphatidylcholine-specific phospholipase D1a (6) Zinc finger protein (7) DNA binding protein FKHL 15 (8) PRAD1
